# Favorable Working Conditions Related to Health Behavior Among Nurses and Care Assistants in Sweden—A Population-Based Cohort Study

**DOI:** 10.3389/fpubh.2021.681971

**Published:** 2021-06-18

**Authors:** Magnus Helgesson, Staffan Marklund, Klas Gustafsson, Gunnar Aronsson, Constanze Leineweber

**Affiliations:** ^1^Division of Insurance Medicine, Department of Clinical Neuroscience, Karolinska Institutet, Stockholm, Sweden; ^2^Department of Psychology, Stockholm University, Stockholm, Sweden

**Keywords:** health complaints, health behavior, physical work conditions, psychosocial work conditions, nurses, care assistants, sick leave, presenteeism

## Abstract

**Objective:** To analyze the associations between favorable physical and psychosocial work factors and health behavior among healthcare employees (nurses and care assistants) with health complaints.

**Methods:** The study was based on seven iterations (2001–2013) of a biennial Swedish work environment survey linked with data from public registers. In all, 7,180 healthcare employees, aged 16–64 years, who had reported health complaints, were included. Health behavior was operationalized through four combinations of sickness absence (SA) and sickness presence (SP): ‘good health behavior' (Low SP/Low SA), ‘recovery behavior' (Low SP/High SA), ‘risk behavior' (High SP/Low SA), and ‘poor health behavior' (High SP/High SA). Odds ratios (OR) were calculated by multinomial logistic regression with 95% confidence intervals (CI).

**Results:** After adjusting for socio-demographic factors, those who rarely worked in strenuous postures had an increased probability of having ‘good health behavior' (OR range: nurses 1.72–2.02; care assistants 1.46–1.75). Those who rarely experienced high job demands had increased odds for having ‘good health behavior' (OR: nurses 1.81; OR range: care assistants 1.67–2.13), while having good job control was found to be related to ‘good health behavior' only among care assistants (OR range 1.30–1.68). In the full model, after also considering differences in health, none of the work environment indicators affected ‘good health behavior' among nursing professionals. Among care assistants, rarely having heavy physical work and having low psychosocial demands remained significantly associated with ‘good health behavior' (OR range: 1.24–1.58) and ‘recovery behavior' (OR range: 1.33–1.70). No associations were found between favorable work environment factors and ‘risk behavior' among the two groups of employees. However, positive assessments of the work situation were associated with ‘good health behavior,' even after controlling for all confounders for both groups (OR range: 1.43–2.69).

**Conclusions:** ‘Good health behavior' and ‘recovery behavior' among care assistants were associated with favorable physical and psychosocial working conditions even when health was considered. This implies that reduced sickness presence and sickness absence among care assistants can be achieved through improved physical and psychosocial working conditions.

## Introduction

Research on factors that may constitute a healthy work environment—one in which people feel well and do not suffer from ill-health—has been more limited than research on risk factors (1–4). A Swedish research review on factors that may support good health shows that fair leadership, teamwork, moderate demands, moderate work pace, and absence of unfavorable physical work conditions enhance employees' health (3). The review found that only one study had focused specifically on analyzing the associations between employees' positive work environment conditions and their health, whereas the remaining studies were concerned with the employees' views of what they believe constitutes a healthy workplace or guidelines for the creation of a healthy workplace (3).

During the last decades, high levels of sickness absence (SA) have been an increasing problem among healthcare employees (5, 6) as well as sickness presence (SP) (7–10). The role of work environment factors for sickness absence and sickness presence has been in the focus of a large number of studies of different occupational groups in different countries (5, 7–16).

Literature reviews and individual studies have also looked at negative social and economic consequences of sickness absence and sickness presence such as loss of income for the worker, increased workload for colleagues, replacement strategies and lower efficiency for the organization and health insurance costs for employer and the society (8, 14, 15, 17–22).

Research on factors that may promote health among healthcare workers have shown that some of the negative effects of sickness absence and sickness presence can be lowered through improved working conditions (2, 3, 11, 23–26). A recent systematic review of interventions intended to reduce occupational stress among healthcare workers concluded that most of the interventions were ineffective, except for programs that included changes in work schedules (26). Another review study, which examined research on factors that foster teamwork among nursing employees, concluded that factors such as motivation, accountability, and support from a supervisor increased the quality of care as well as employee satisfaction (24). Similarly, a study on the research on nursing leaders concluded that work environments could be improved through effective communication, collaborative relationships, and increased decision-making responsibility among the nurses (23). Unfortunately, none of these reviews focused on the associations between psychosocial or physical work environment factors and the employees' health or health behavior.

In one of the few studies that tried to determine whether the presence or absence of certain work environment determinants were associated with health, it was found that some physical and psychosocial factors tended to be associated with both good health and poor health depending on the positive or negative value of the indicator. For example, absence of heavy lifting and good job support were associated with good health while frequent exposure to heavy lifting and lack of job support were associated with poor health. Other factors were found to have associations only at one side of the health continuum. Thus, high role clarity increased the probability of good health, while low role clarity was not associated with poor health (2).

One of the reasons why studies on factors that contribute to good health reach different results is that they are based on differing perspectives and definitions of health. Mainly two perspectives have dominated research. The biomedical perspective focuses on illness and its symptoms, while the behavioral perspective focuses on actions of the individual where health-related restrictions, such as low work ability or sickness absence, are central (27). Although correlations have been found between different self-reported general ill-health variables derived from different concepts, and both sickness absence and sickness presence, there are also differences between them. One such difference is that the number of individuals who report ill-health is much larger than the numbers of individuals with a diagnosed disease or who are registered as sickness absent (28).

The present study focused on differences in working conditions and health behavior among employees who had reported health complaints. Not all reported complaints are likely to be regarded as a work limitation by employees, either because the associated problems are limited in relationship to the person's work obligations or because effective medication has recreated normal work ability. It is also likely that employees with more favorable working conditions would go to work despite health symptoms as they are likely able to adjust their work obligations to their health limitations and perceive their work as giving. Those who are exposed to unfavorable physical or psychosocial work conditions may have difficulties to adjust their work obligations to their health and thus be more likely to report sickness absence or sickness presence. We thus assume that differences in working conditions that could impact employees' health are related to their health behavior.

Health behavior can be defined as behavioral patterns, actions, and habits that relate to health maintenance, to health restoration and to health improvement of the individual (29). One specific aspect of health behavior concerns peoples' decision to go to work or not when they have a health problem. If they abstain from work, they are sickness absent, and if they go to work despite their health conditions, they are sickness present. Both the severity and character of the health problems as well as working conditions might influence on employees' health behavior.

The present study examined health behavior classified in four combinations of sickness presence (SP) or sickness absence (SA) among healthcare employees who had reported some health complaints, namely ‘good health behavior' (low SP/low SA), ‘recovery behavior' (low SP/high SA), ‘risk behavior' (high SP/low SA), and ‘poor health behavior' (high SP/high SA). Employees in the ‘good health behavior' group may have limited health problems or may have health problems that do not affect their work ability, while individuals who were classified as engaging in ‘recovery behavior' were sickness absent when experiencing ill health. Those engaging in ‘risk behavior' were present at work although their health condition in their own view should have led to sickness absence. We assumed that sickness absence, as shown in the ‘good health behavior' and ‘recovery behavior' group, might be more likely to facilitate recovery than sickness presence. This is also an assumption that has partly been confirmed in a recently published study (22).

The focus of the present study is on favorable work environment aspects. Some often-used psychosocial indicators include positive as well as negative aspects, where, for example, the response options to an item on job control include answers indicating a high degree of control as well as not having control. Other indicators have primarily been used to measure negative aspects, such as the measure of strenuous positions, which only includes the existence of disadvantageous conditions. In the present study the absence of such negative factors is seen as indicating favorable working conditions.

The main aim of the present study was to investigate the associations between indicators of favorable physical and psychosocial working conditions and health behavior among nursing professionals and care assistants in Sweden who reported health complaints. Four combinations of sickness absence and sickness presence were used to represent different health behaviors. A second aim was to identify differences and similarities between nursing professionals and care assistants regarding the degrees to which different work factors contributed to good health behavior.

## Methods

This cross-sectional population-based study utilized data on seven cohorts of the Swedish employed population collected from surveys between 2001 and 2013. The study population consisted of employees in health and social care occupations that had reported health complaints.

### Data Sources

Information about the employed individuals that was used in this study originated from two different data registers: the Swedish Work Environment Surveys (SWES) and the population-based Longitudinal Integrated Database for Health Insurance and Labor Market Studies (LISA). The prime data source were the biennial Swedish Work Environment Surveys SWES, which has been developed by Statistics Sweden (SCB) on behalf of the Swedish Work Environment Authority. The SWES has been conducted every second year since 1989. The surveys were administered to random samples of the Swedish employed population aged 18–64 through telephone interviews and a supplementary postal questionnaire by Statistics Sweden. An official translation of the survey questionnaire into English are presented in Supplementary File. There were in total 126 items in the survey and for this study we used 30 items. All responses to the questions were self-reported and response alternatives were Likert scales (30). In the present study, data from 7,180 healthcare employees from seven iterations of the surveys between 2001 and 2013 were included. The response rates for the surveys varied between 77 and 66%. The data covers a broad range of physical and psychosocial working conditions (30, 31).

Data on individual background factors and annual information on sickness absence for the period from 2001 to 2013 were attained from the Longitudinal Integrated Database for Health Insurance and Labor Market Studies (LISA) database at Statistics Sweden (SCB). This includes data on participants' medically certified sickness absence periods lasting at least 15 days, that is, long enough for compensation from the national social insurance system to apply.

### Classification of Occupations and Selection of Participants

The two occupational categories of prime interest in this study consist of employees in health and social care, classified according to the 1996 Swedish Standard Classification of Occupations (SSYK-96). The Standard for Swedish Occupational Classification (SSYK-96) at the three-digit level was used for identifying different types of occupations. SSYK-96 closely follows the International Standard Classification of Occupations, ISCO-88, from the ILO, which is used in the statistical publications from the European Union.

Two subgroups of health and social care employees were used. The first, “Nursing professionals,” (*n* = 1,373) included nursing and midwifery professionals and nursing associate professionals (SSYK-96, code 223 and SSYK-96, code 323) with a University degree and who were working in hospitals or other health care organizations. The second subgroup, “Care assistants,” (*n* = 5,807) consisted of employees in personal care and related workers such as assistant nurses, also including hospital ward assistants, home-based personal care workers, and childcare assistants (SSYK-96, code 513). The educational requirement for these occupations was generally upper-secondary education. In the final study population, data on 7,180 nurses and care assistants were used.

### Measurements

#### Health Complaints

As the study concerned health behavior among employees, a selection of participants with health complaints was made based on the following question in the SWES survey. “Have you during the past 3 months after work suffered from any of the following?” (Number and %).

- Had pain in upper back or neck 2,659 (38%)
- Had pain in lower back 3,142 (45%)
- Had pain in shoulders or arms 2,417 (35%)
- Had pain in wrists or hands 3,171 (46%)
- Been physically tired 3,595 (50%)
- Been tired and listless 2,241 (32%)
- Had headache 2,822 (39%)
- Had itchy or otherwise irritated eyes 2,969 (42%)
- Had trouble sleeping 2,006 (28%)

The response alternatives were: “Yes, every day,” “Yes, 1 day of 2,” “Yes, 1 day of 5,” “Yes, 1 day of 10,” and “No, Not at all or rarely in the last 3 months.” In this study, exposure to any of the health complaints was equal to the upper quartile of exposure in the entire population in the SWES survey.

#### Sickness Absence

In Sweden, the general regulations regarding sickness absence compensation have only changed marginally during the period covered in this study (32). The first day of sickness absence is a non-compensated waiting day, while days 2 through 14 are covered by employer compensation. From day 15 on, sickness absence compensation is paid by the National Social Insurance Agency. By the 8th day, a medical certification of the limitation in work ability is required. Here, we considered the total number of net days of compensated sickness absence over the calendar year in which the individual participated in the SWES survey was calculated based on data from the LISA database. Individuals without compensated sickness absence from the social security system, that is <14 days of total sickness absence, were defined as “low” sickness absence (*n* = 5,808, 80.9%), while those who had one or more than 1 day of compensated sickness absence, that is, at least 15 days of total sickness absence, were considered as having “high” sickness absence (*n* = 1,372, 19.1%).

#### Sickness Presence

The number of times of self-reported sickness presence was measured in the SWES survey by the item “Has it happened over the previous 12 months that you have gone to work despite feeling that you really should have taken sick leave because of your state of health?,” using a four-point response scale [Never (*n* = 1,590; 22.1%); Yes, one occasion (*n* = 1,354; 18,9 %), Yes, two-three occasions (*n* = 2,657; 37.0%), Yes, four or more occasions (*n* = 1,579: 22.0%)]. Further, sickness presenteeism behavior was dichotomized into high (two or more occasions) and low (never or once).

#### Health Behavior

Health behavior was the outcome measure. In this study, four combinations of high and low sickness presence (SP) and sickness absence (SA) were used to characterize differences in the health behavior of the participants. The operationalization of the four combinations were:

- ‘good health behavior' (low SP/low SA)
- ‘recovery behavior' (low SP/high SA)
- ‘risk behavior' (high SP/low SA)
- ‘poor health behavior' (high SP/high SA, reference category).

#### Work Environment Variables

Several favorable work environment factors were used as exposure variables in the study. Data on physical and psychosocial working conditions were obtained from SWES for the period 2001–2013 (30). The present study used favorable values on the indicators of the work environment. For the items concerning heavy physical work, strenuous postures, exposure to substances, and high psychosocial job demands, favorable values were those indicating no-exposure, while for the items concerning job control, support from colleges, and support from supervisors, the advantageous values were derived from the beneficial side of the response alternatives, that is, having good control and good support.

Three items were chosen as indicators of not having heavy physical work. The response scales were dichotomized closest to the upper quartile of the response alternatives. Responses indicating favorable conditions are given in parenthesis.

1. Does your job mean that your work is purely physical, i.e., do you put in more physical effort than you do when you walk, stand and move in the usual way? (No, not at all)
2. Are you required to lift at least 15 kg at a time several times per day? (No, not at all)
3. Do you exert yourself so much that you breathe faster? (No, not at all)

The three items below were chosen as indicators of not having strenuous work postures. The response scales were dichotomized closest to the upper quartile of the response alternatives. Responses indicating favorable conditions are given in parenthesis.

1. Do you work bent forward, without supporting yourself with your hands or arms? (No, not at all)
2. Do you work in a twisted position? (No, not at all)
3. Do you bend or twist yourself in your work in the same way repeatedly in an hour, for several hours during the same day? (No, not at all)

Three items were chosen as indicators of exposure to substances (biological or chemical risk factors). The response scales were dichotomized closest to the upper quartile of the response alternatives to identify individuals who were not exposed. Responses indicating favorable conditions are given in parenthesis.

1. Are you exposed to any of the following in your work?
2. Detergents and/or disinfectants (in contact with the skin)? (No, not at all)
3. Water that comes in direct contact with the skin several times per hour (including when washing)? (No, not at all)
4. Human secretions such as saliva, blood, urine, feces, or vomit? (No, not at all)

Data on psychosocial working conditions were also obtained from the SWES surveys (30). In line with previous research by Magnusson Hanson and collaborators (33), job demands, job control, and job support were measured using a number of items which served as proxy indicators of the demand-control model as formulated by Karasek and collaborators (33, 34). The items for each scale were summarized and dichotomized closest to the upper quartile of the response alternatives. Responses indicating favorable conditions are given in parenthesis.

The following three items captured job demands:

1. Is your work so stressful that you do not have time to talk or even think about something other than work? (No, not at all or about 1/10 of the time)
2. Does the work require your full attention and concentration? (No, not at all, about 1/10 of the time or about 1/4 of the time)
3. Do you have so much work that you must miss lunch, work late or take work home? (No, not at all)

Three items were used to capture aspects of job control. The responses indicating favorable conditions are given in parenthesis:

1. Do you have the opportunity to determine your work pace? (Yes, nearly all the time, about 3/4 of the time)
2. Are you able to determine when various working duties are to be carried out (for example, by choosing to work a bit faster on some days and taking it easier on other days)? (Yes, always or Yes, mostly)
3. Do you participate in decisions on the arrangement of your work (e.g., what is to be done, how to do it or who will work with you) (Yes, always)

Two items concerned job support from supervisors or fellow workers and responses indicating favorable conditions are given in parenthesis.

1. Are you able to get support and encouragement from supervisors when work feels difficult? (Yes, always)
2. Are you able to get support and encouragement from colleges when work feels difficult? (Yes, always)

#### Work Assessments

To complement the abovementioned work environment factors, three items concerning the employees' subjective work assessments were also used. The response scales were dichotomized closest to the upper quartile of the response alternatives. These responses were seen to indicate that the employees did not report negative feelings about their work. Responses indicating favorable conditions are given in parenthesis.

1. At the end of your workday, do you feel that your work input is inadequate? (No, not at all)
2. Do you feel anxiety when you go to work? (No, not at all)
3. Do you feel ill at ease and downhearted as a result of the difficulties you face at work? (No, not at all)

#### Confounders

Several factors, which have been found to be relevant in previous research on health behavior, were used as confounders in the study. These include socio-economic factors such as sex, age at time of interview (divided into 18–30, 31–40, 41–50, and 51–64 years), number of years of formal education (divided into ≤9 years, 10–12 years, and >12), and employment sector (public sector or private sector). This information was obtained from the LISA database.

Further, health status was used as an additional confounder to assess to what degree the associations between health behavior and working conditions were affected by differences in health status. Health status was measured by the above-described question concerning health complaints that was also used for selecting the study group and obtained from the SWES database. When used as a confounder, an additive index called “the health complaints index” was constructed. Each of the 5 responses to the nine items was recoded into values 1–5, where 1 represented no or low and 5 represented having pain every day. Thus, the value of the index varied between 9 and 45. The index was used in this continuous format.

### Statistical Analyses

The analyses were conducted in two steps. In the first step, descriptive statistics regarding occupational group and socio-demographic characteristics (sex, age, education) were analyzed in relation to the four combinations of sickness presence and sickness absence (‘good health behavior,' ‘recovery behavior,' ‘risk behavior,' and ‘poor health behavior') (Table 1). Additionally, prevalences for nursing professionals and care assistants of being in either of the four behavior categories were calculated for the work environment indicators (Table 2).

**Table 1:** Distribution of the four health behavior groups (‘good health behavior,' ‘recovery behavior,' ‘risk behavior' and ‘poor health behavior') according to occupation, sex, age, education, employment sector, and health complaints, 2001–2013^a^.

	**Good health behavior**	**Recovery behavior**	**Risk behavior**	**Poor health behavior**
	**Low SP and Low SA**	**Low SP and High SA**	**High SP and Low SA**	**High SP and High SA**
	***N* = 2,573**	***N* = 371**	***N* = 3,235**	***N* = 1,001**
**Variables^b^**	***n***^**c**^ **(*****P***^**d**^**)**	***n*****^c^** **(*****P*****^d^)**	***n*****^c^** **(*****P*****^d^)**	***n*****^c^** **(*****P*****^d^)**
**Occupation**				
Nursing professionals	563 (41%)	69 (5%)	576 (42%)	165 (12%)
Care assistants	2,010 (35%)	302 (5%)	2,659 (46%)	836 (14%)
**Sex**				
Men	239 (38%)	19 (3%)	313 (50%)	51 (8%)
Women	2,334 (36%)	352 (5%)	2,922 (45%)	950 (14%)
**Age at interview (years)**				
18–30	319 (35%)	23 (3%)	474 (52%)	93 (10%)
31–40	487 (32%)	70 (5%)	774 (51%)	193 (13%)
41–50	734 (35%)	109 (5%)	980 (46%)	299 (14%)
51–64	1,033 (39%)	169 (6%)	1,007 (38%)	416 (16%)
**Education (years)**				
>12	145 (33%)	26 (6%)	192 (44%)	74 (17%)
10–12	1,444 (34%)	233 (5%)	1,930 (46%)	635 (15%)
<9	984 (39%)	112 (4%)	1,113 (45%)	292 (12%)
**Employment sector**				
Private	545 (40%)	58 (4%)	625 (45%)	146 (11%)
Public	2,020 (35%)	311 (5%)	2,604 (45%)	852 (15%)
**Health complaints index**				
9–15 points	825 (32%)	79 (21%)	494 (15%)	92 (9%)
16–30 points	1,610 (63%)	252 (68%)	2,274 (70%)	641 (64%)
31–45 points	138 (5%)	40 (11%)	467 (14%)	268 (27%)

*Percent distribution in parenthesis*.

a *All n = 7,180*.

b *For all variables, the chi^2^ tests are significant: p < 0.001*.

c *Number of cases (n)*.

d *Prevalence (P) of the exposure categories (%)*.

**Table 2:** Prevalences among the four behavior groups (‘good health behavior,' ‘recovery behavior,' ‘risk behavior,' and ‘poor health behavior') according to favorable working conditions among nursing professionals and care assistants, 2001–2013^a^.

	**Nursing professionals (*****n*** **=** **1,373)**								**Care assistants (*****n*** **=** **5,807)**							
**Favorable work environment factors^d^**	**Good health behavior**		**Recovery behavior**		**Risk behavior**		**Poor health behavior**		**Good health behavior**		**Recovery behavior**		**Risk behavior**		**Poor health Behavior**	
	***n*^b^**	***P*^c^**	***n*^b^**	***P*^c^**	***n*^b^**	***P*^c^**	***n*^b^**	***P*^c^**	***n*^b^**	***P*^c^**	***n*^b^**	***P*^c^**	***n*^b^**	***P*^c^**	***n*^b^**	***P*^c^**
**Heavy physical work**																
Not working only physically	177	38	23	38	173	36	53	39	477	28	66	26	504	23	150	22
No heavy lifting	367	66	43	62	337	59	101	62	1,070	54	152	51	1,114	42	319	38
No exertion until breathing fast	348	74	45	74	309	63	91	66	1,037	61	144	56	1,036	47	276	41
**Strenuous work postures**																
Not working bent forward	172	37	21	34	146	30	34	25	562	33	78	30	580	26	160	24
Not working in a twisted position	144	31	10	17	116	24	30	22	510	30	55	22	514	23	132	19
Not bent or twisting repeatedly	346	62	43	62	313	54	92	56	940	47	132	44	973	37	272	33
**Exposure to substances**																
No detergents or disinfectants	245	44	27	39	191	33	56	34	858	43	134	45	1,025	39	328	40
No water in contact with skin	134	24	14	20	109	19	29	18	591	30	85	28	700	27	212	26
No human secretions	122	22	12	17	90	16	28	17	570	29	82	28	667	25	172	21
**Job Demands**																
Stressful, no time to think <1/10 of time	166	30	21	31	106	19	32	20	918	46	137	46	858	33	242	29
Attention/concentration < $\frac{1}{2}$ of time	107	19	7	10	64	11	24	15	482	24	69	23	520	20	132	16
Not much work (miss lunch, work late, take home)	122	22	20	29	81	14	31	19	1,058	53	154	52	1,038	39	311	38
**Job Control**																
Determine work pace ≥3/4 of time	145	26	22	32	111	20	33	20	680	34	106	35	735	28	192	23
Always or mostly determine working duties	179	32	13	19	151	27	43	26	701	35	106	35	873	33	235	28
Always participate in decisions	125	22	12	17	111	20	26	16	419	21	60	20	522	20	138	17
**Job support**																
Always from supervisors	81	15	14	20	60	11	11	7	411	21	63	21	357	14	135	16
Always from colleagues	195	35	26	38	176	31	43	26	884	45	136	45	999	38	315	38
**Employees' assessments of their work**																
My work input is not at all inadequate	178	37	22	44	131	27	25	20	849	50	121	50	809	37	211	33
I do not at all feel anxiety when I go to work	366	65	47	68	261	46	48	29	1,332	67	185	62	1,179	45	346	42
I do not at all feel ill at ease due to difficulties at work	256	53	29	58	196	40	35	28	1,118	66	158	66	1,040	47	287	45

*Percent distribution*.

a *All n = 7,180*.

b *Number of individuals to have reported a positive value (n)*.

c *Prevalence (P) of the exposure categories, a positive value (%)*.

d *Results show work conditions for the best quartile of the response scale on each item. The exact wordings of the items are presented in the methods section*.

In the second step, multiple multinomial logistic regression analyses were conducted to determine the predictive values of data concerning working conditions. Multinomial logistic regression analyses were used because the outcome variable had four values, defined as the four different health behavior categories. The regression analyses were conducted with two different models, where one estimation of odds ratios included adjustments for the socio-economic confounders, year of interview, age at interview, sex, education, and employment sector (Model I). The other model consisted of calculations of odds ratios, where the health complaints index was introduced as an additional confounder (Model II). In both models, the analyses were stratified for nursing professionals and care assistants, respectively. The reference category used in all the multinomial regressions was ‘poor health behavior.' The results are presented in Tables 3, 4, including odds ratios and 95% confidence intervals. All statistical analyses were conducted with SAS, version 9.4, statistical software (SAS Institute, Inc., Cary, North Carolina).

**Table 3:** Adjusted odds ratios (OR) and 95% confidence intervals (CI) for the ‘good health behavior,' ‘recovery behavior,' and ‘risk behavior' combinations among nursing professionals (*n* = 1,373), 2001–2013, compared with ‘poor health behavior' (reference category, OR = 1) in relation to favorable working conditions.

	**Nursing professionals**					
	**Model I**			**Model II**		
**Favorable work environment factors^b^**	**Good health Behavior**	**Recovery behavior**	**Risk behavior**	**Good health Behavior**	**Recovery behavior**	**Risk behavior**
	**OR^a^ (95% CI)**	**OR^a^ (95% CI)**	**OR^a^ (95% CI)**	**OR^a^ (95% CI)**	**OR^a^ (95% CI)**	**OR^a^ (95% CI)**
**Heavy physical work**						
Not working only physically	1.06 (0.70–1.61)	1.00 (0.52–1.92)	1.06 (0.70–1.60)	0.84 (0.54–1.30)	0.80 (0.41–1.56)	0.96 (0.63–1.47)
No heavy lifting	1.21 (0.83–1.76)	0.99 (0.54–1.81)	0.96 (0.66–1.40)	0.87 (0.58–1.30)	0.74 (0.40–1.39)	0.85 (0.58–1.25)
No exertion till breathing fast	**1.60 (1.04–2.46)**	1.56 (0.78–3.14)	1.04 (0.68–1.59)	1.13 (0.72–1.79)	1.14 (0.56–2.34)	0.91 (0.59–1.41)
**Strenuous work postures**						
Not working bent forward	**2.02 (1.28–3.17)**	1.61 (0.81–3.19)	**1.60 (1.01–2.53)**	1.53 (0.95–2.47)	1.25 (0.62–2.52)	1.45 (0.91–2.31)
Not working in a twisted position	**1.72 (1.07–2.74)**	0.72 (0.32–1.63)	1.29 (0.80–2.07)	1.26 (0.77–2.07)	0.54 (0.24–1.24)	1.14 (0.70–1.85)
Not bent or twisting repeatedly	1.32 (0.92–1.88)	1.31 (0.74–2.35)	1.01 (0.71–1.44)	0.84 (0.57–1.23)	0.88 (0.48–1.61)	0.83 (0.57–1.20)
**Exposure to substances**						
No detergents or disinfectants	**1.60 (1.10–2.34**)	1.18 (0.65–2.17)	1.07 (0.73–1.57)	1.25 (0.84–1.86)	0.94 (0.50–1.74)	0.97 (0.66–1.44)
No water in contact with skin	1.38 (0.87–2.18)	1.09 (0.52–2.26)	1.08 (0.68–1.72)	0.97 (0.60–1.57)	0.79 (0.38–1.67)	0.93 (0.57–1.49)
No human secretions	1.43 (0.89–2.29)	0.96 (0.45–2.06)	1.02 (0.63–1.65)	1.04 (0.64–1.71)	0.72 (0.33–1.57)	0.88 (0.54–1.43)
**Job Demands**						
Stressful, no time to think <1/10 of time	**1.81 (1.17–2.80)**	1.81 (0.94–3.49)	0.94 (0.60–1.47)	1.25 (0.79–1.98)	1.33 (0.68–2.61)	0.81 (0.51–1.29)
Attention/concentration < half of time	1.31 (0.80–2.14)	0.63 (0.25–1.56)	0.67 (0.40–1.13)	0.96 (0.57–1.60)	0.47 (0.19–1.19)	**0.58 (0.34–0.98)**
Not much work (miss lunch, work late, take home)	1.24 (0.79–1.95)	1.78 (0.92–3.47)	0.75 (0.47–1.19)	1.07 (0.67–1.72)	1.57 (0.80–3.09)	0.70 (0.43–1.12)
**Job control**						
Determine work pace ≥3/4 of time	1.43 (0.93–2.21)	1.84 (0.97–3.51)	1.03 (0.66–1.60)	1.14 (0.72–1.80)	1.51 (0.78–2.93)	0.94 (0.60–1.48)
Always or mostly determine working duties	1.37 (0.92–2.05)	0.63 (0.31–1.27)	1.08 (0.72–1.62)	1.06 (0.70–1.61)	0.49 (0.24–1.01)	0.97 (0.64–1.46)
Always participate in decisions	1.56 (0.98–2.50)	1.13 (0.53–2.41)	1.33 (0.83–2.14)	1.36 (0.84–2.22)	1.01 (0.47–2.17)	1.25 (0.77–2.02)
**Job support**						
Always from supervisors	**2.34 (1.20–4.58)**	**3.44 (1.45–8.18)**	1.49 (0.75–2.95)	1.68 (0.84–3.35)	**2.57 (1.06–6.24)**	1.31 (0.65–2.61)
Always from colleges	**1.48 (1.00–2.21)**	1.78 (0.97–3.28)	1.16 (0.78–1.74)	1.20 (0.80–1.83)	1.48 (0.80–2.76)	1.08 (0.72–1.62)
**Employees' assessments of their work**						
My work input is not at all inadequate	**2.17 (1.34–3.51)**	**2.91 (1.41–5.99)**	1.43 (0.88–2.34)	1.57 (0.95–2.58)	2.07 (0.99–4.34)	1.25 (0.76–2.05)
I do not at all feel anxiety when I go to work	**4.43 (3.02–6.50)**	**5.19 (2.81–9.57)**	**2.06 (1.41–3.02)**	**2.69 (1.79–4.05)**	**3.47 (1.83–6.57)**	**1.72 (1.16–2.57)**
I do not at all feel ill at ease due to difficulties at work	**2.79 (1.80–4.31)**	**3.42 (1.71–6.86)**	**1.68 (1.09–2.60)**	**1.90 (1.20–2.99)**	**2.31 (1.13–4.73)**	1.47 (0.94–2.30)

*Calculated with multinomial logistic regression*.

a *Odds ratio (OR) with 95% confidence interval (CI). Model I, adjusted for sex, age, education, employment sector, and year of interview. Model II, additionally adjusted for health complaints index. Bold, statistically significant at the p < 0.05 level*.

b *Results show work conditions for the best quartile of the response scale on each item. The exact wordings of the items are presented in the methods section*.

**Table 4:** Adjusted odds ratios (OR) and 95% confidence intervals (CI) for the ‘good health behavior,' ‘recovery behavior,' and ‘risk behavior' combinations among care working assistants (*n* = 5,807), 2001–2013, compared with ‘poor health behavior' (reference category, OR = 1) in relation to favorable conditions.

	**Care assistants**					
	**Model I**			**Model II**		
**Favorable work environment factors^b^**	**Good health behavior**	**Recovery Behavior**	**Risk behavior**	**Good health Behavior**	**Recovery behavior**	**Risk behavior**
	**OR^a^ (95% CI)**	**OR^a^ (95% CI)**	**OR^a^ (95% CI)**	**OR^a^ (95% CI)**	**OR^a^ (95% CI)**	**OR^a^ (95% CI)**
**Heavy physical work**						
Not working only physically	1.22 (0.98–1.51)	1.22 (0.86–1.73)	0.98 (0.79–1.22)	0.86 (0.68–1.09)	0.96 (0.68–1.37)	0.85 (0.68–1.06)
No heavy lifting	**1.76 (1.48–2.08)**	**1.64 (1.25–2.16)**	1.15 (0.97–1.36)	**1.24 (1.03–1.48)**	**1.33 (1.00–1.75)**	1.01 (0.85–1.19)
No exertion till breathing fast	**2.14 (1.78–2.57)**	**1.81 (1.35–2.43)**	**1.28 (1.07–1.53)**	**1.44 (1.18–1.75)**	**1.38 (1.02–1.88)**	1.10 (0.91–1.32)
**Strenuous work postures**						
Not working bent forward	**1.46 (1.18–1.80)**	**1.43 (1.03–1.99)**	1.08 (0.88–1.33)	0.99 (0.79–1.24)	1.10 (0.79–1.55)	0.93 (0.75–1.15)
Not working in a twisted position	**1.62 (1.30–2.03)**	1.17 (0.81–1.69)	1.18 (0.95–1.48)	1.10 (0.87–1.39)	0.88 (0.61–1.28)	1.02 (0.81–1.28)
Not bent or twisting repeatedly	**1.75 (1.47–2.09)**	**1.60 (1.21–2.10)**	1.16 (0.98–1.38)	1.10 (0.91–1.32)	1.20 (0.90–1.60)	0.98 (0.82–1.16)
**Exposure to substances**						
No detergents or disinfectants	1.06 (0.89–1.26)	1.24 (0.94–1.63)	0.93 (0.79–1.10)	0.90 (0.75–1.08)	1.13 (0.85–1.49)	0.89 (0.75–1.05)
No water in contact with skin	1.01 (0.83–1.23)	1.15 (0.84–1.57)	0.92 (0.76–1.11)	0.81 (0.66–0.99)	1.00 (0.73–1.38)	0.85 (0.70–1.03)
No human secretions	**1.28 (1.04–1.57)**	**1.51 (1.09–2.09)**	1.15 (0.94–1.41)	1.12 (0.90–1.40)	**1.40 (1.01–1.95)**	1.11 (0.90–1.36)
**Job Demands**						
Stressful, no time to think <1/10 of time	**2.13 (1.78–2.53)**	**2.11 (1.60–2.78)**	1.18 (0.99–1.41)	**1.47 (1.22–1.78)**	**1.70 (1.29–2.26)**	1.03 (0.86–1.23)
Attention/concentration < half of time	**1.67 (1.35–2.07)**	**1.61 (1.16–2.24)**	**1.28 (1.03–1.58**)	**1.42 (1.13–1.78)**	**1.45 (1.04–2.03)**	1.20 (0.97–1.49)
Not much work (miss lunch, work late, take home)	**2.00 (1.69–2.37)**	**1.77 (1.35–2.32)**	1.14 (0.97–1.34)	**1.58 (1.32–1.88)**	**1.52 (1.16–2.00)**	1.04 (0.88–1.23)
**Job Control**						
Determine work pace ≥3/4 of time	**1.68 (1.39–2.03)**	**1.87 (1.40–2.50)**	**1.27 (1.06–1.53)**	**1.40 (1.15–1.71)**	**1.68 (1.26–2.25)**	1.19 (0.99–1.44)
Always or mostly determine working duties	**1.31 (1.09–1.56)**	**1.42 (1.07–1.89)**	**1.20 (1.01–1.43)**	1.12 (0.92–1.35)	**1.30 (0.98–1.74)**	1.15 (0.96–1.37)
Always participate in decisions	**1.30 (1.05–1.61)**	1.26 (0.89–1.76)	**1.23 (1.00–1.52)**	1.23 (0.98–1.54)	1.23 (0.87–1.73)	1.22 (0.99–1.51)
**Job support**						
Always from supervisors	**1.32 (1.07–1.64)**	**1.40 (1.00–1.96)**	**0.79 (0.64–0.98)**	1.09 (0.87–1.37)	1.26 (0.89–1.77)	**0.74 (0.60–0.93)**
Always from colleges	**1.39 (1.17–1.65)**	**1.40 (1.07–1.84)**	1.00 (0.85–1.18)	1.16 (0.97–1.38)	1.25 (0.95–1.65)	0.93 (0.79–1.10)
**Employees' assessment of their work**						
My work input is not at all inadequate	**2.05 (1.69–2.49)**	**2.04 (1.50–2.77)**	1.16 (0.96–1.40)	**1.43 (1.16–1.75)**	**1.60 (1.17–2.18)**	1.01 (0.83–1.23)
I do not at all feel anxiety when I go to work	**2.89 (2.44–3.43)**	**2.17 (1.65–2.85)**	**1.20 (1.02–1.41)**	**1.75 (1.46–2.09)**	**1.59 (1.20–2.12)**	0.97 (0.82–1.15)
I do not at all feel ill at ease due to difficulties at work	**2.49 (2.06–3.01)**	**2.30 (1.68–3.14)**	1.13 (0.94–1.35)	**1.60 (1.31–1.95)**	**1.71 (1.24–2.36)**	0.94 (0.78–1.14)

*Calculated with multinomial logistic regression*.

a *Odds ratio (OR) with 95% confidence interval (CI). Model I, adjusted for sex, age, education, employment sector, and year of interview. Model II, additionally adjusted for health complaints index. Bold, statistically significant at the p < 0.05 level*.

b *Results show work conditions for the best quartile of the response scale on each item. The exact wordings of the items are presented in the methods section*.

## Results

Table 1 gives an overview of the distribution of the study population into the four health behavior categories, ‘good health behavior,' ‘recovery behavior,' ‘risk behavior,' and ‘poor health behavior,' according to occupation, gender, age, education, and employment sector. Just over 40 percent of the nursing professionals (41%) and somewhat fewer of the care assistants (35%) qualified for the ‘good health behavior' category (Table 1). Among both occupational groups, only 5% were categorized as qualified for the ‘recovery behavior' group while a lower proportion among care assistants than among nurses qualified for the ‘risk behavior' category (46 and 42%, respectively). A lesser proportion of the nurses were classified as practicing ‘poor health behavior' (12%) compared to care assistants (14%).

It can also be seen in Table 1 that somewhat larger proportions of men than women qualified for being classified in the ‘good health behavior' group. Larger proportions among those with tertiary education than among those with shorter education, and among those in the private than the public sector, also had ‘good health behavior.' Larger proportions of people aged over 50 fell into the ‘poor health behavior' group than young people (Table 1). It is also shown in Table 1 that employees in the ‘good health behavior' group reported fewer or less frequent health complaints when compared to the other three groups. About 1/3 of the ‘good health behavior' group, 21% of the ‘recovery behavior' group, 15% of the ‘risk behavior' group, and 9% of the ‘poor health behavior' group had the lowest number of complaints (Table 1).

Table 2 presents the pre-valences of the four health behavior combinations among nursing professionals and care assistants reporting various physical and psychosocial working conditions. Among both nurses and care assistants, with few exceptions, higher proportions of employees with favorable physical working conditions were in the ‘good health behavior' category as compared to those exposed to heavy physical work, strenuous work postures, or substances. A similar pattern was shown for the associations between two of the three indicators of favorable psychosocial conditions, as larger proportions among those with low job demands and high job control were also in the ‘good health behavior' category. The associations between the indicators of good job support and the four combinations were more complex. Nurses as well as care assistants who reported that they always could get support from their supervisors or colleagues showed the highest proportion for the ‘recovery behavior' category, but for both occupational groups, the pre-valences of ‘risk behavior' and ‘poor health behavior' were lower among those who could get support (Table 2).

With regard to employees' assessments about their work, the results show that a positive work assessment not only goes along with higher pre-valences for both ‘good health behavior' and ‘recovery behavior,' but also with lower pre-valences for ‘risk behavior' and ‘poor health behavior.' It should also be noted that the proportion in the ‘good health behavior' category was very high among those nursing professionals and care assistants who reported positive assessments about their work.

Table 3 presents the adjusted odds ratios based on multinomial logistic regression analyses of the effects of the favorable indicators of physical and psychosocial working conditions among nursing professionals on three of the health categories.

For nursing professionals, no ‘exertion until breathing fast' significantly increased the probability of ‘good health behavior' in the controlled model (OR 1.60; Table 3, Model I). Not having any strenuous work postures increased the odds of ‘good health behavior' between 68 and 98% for two of the indicators. Similarly, not being exposed to detergents or disinfectants increased the probability ‘good health behavior' among nurses (OR 1.60). One of the indicators of low job demands increased the probability of ‘good health behavior' (job not stressful OR 1.81). All indicators of high job control significantly increased the probability, by between 52 and 78%, for ‘good health behavior' among nursing professionals, and the indicators of support from supervisors showed increased odds for ‘good health behavior' (OR 2.34; Table 3, Model I).

High odds for being classified in the ‘good health behavior' group among nurses were found for the factors concerning employees' assessments and feelings about their work (OR between 2.17 and 4.43) in the model where socio-economic differences were controlled for (Table 3, Model I).

As expected, the odds ratios for ‘good health behavior' were lower when additional adjustment was made for health differences among the participants (Table 3, Model II).

Among nurses, a number of the advantageous working conditions that increased their odds for ‘good health behavior,' such as absence of heavy work, no strenuous postures, low psychosocial demands, and availability of job support, were no longer significant when the index for health complaints was introduced as a confounder, indicating that health complaints were a predictor of health behavior. However, two of the three factors concerning employees' general assessments about their job remained significantly associated with ‘good health behavior' even after controlling for health differences (OR between 1.90 and 2.69) (Table 3, Model II).

Among care assistants, low exposure to two of the three indicators of heavy physical work (OR 1.76, OR 2.14, respectively), and to all three indicators of low exposure to strenuous work positions (OR 1.46, OR 1.62, OR 1.75, respectively), were significantly related to the ‘good health behavior' group (Table 4, Model I).

Similarly, there was a high probability for care assistants with low job demands, high job control, and good job support to be in the ‘good health behavior' category (ORs between 1.30 and 2.13, for the different indicators) (Table 4, Model I). Individuals reporting high levels of any of these indicators of favorable working conditions had a greater probability of being in the “recovery behavior' group”, although the odds ratios were somewhat lower. Among care assistants, high odds were also found for the three indicators of employees' work assessments in relation to the ‘good health behavior' category (ORs between 2.05 and 2.89; Table 4, Model I).

As compared to nurses, the odds ratios for ‘good health behavior' were less changed among care assistants when additionally adjusting for the health complaints index. Notably increased odds for ‘good health behavior' were found for care assistants who reported not being exposed to heavy physical work (OR 1.24 and OR 1.44, respectively) and who reported low psychosocial demands (OR 1.47, OR 1.42, OR 1.58, respectively) (Table 4, Model II). There were similar, or even stronger, associations between these factors and ‘recovery behavior' among care assistants.

The main difference between nursing professionals and care assistants is in the specification of relevant favorable work environment factors affecting ‘good health behavior.' None of the studied physical or psychosocial factors were associated with ‘good health behavior' among nurses, whereas low exposure to heavy work, low job demands, and ability to determine the work place all increased the probability of ‘good health behavior' and ‘recovery behavior' among care assistants. However, for both occupational groups, those who had a positive general assessment of their work were also more likely to practice ‘good health behavior' or ‘recovery behavior.'

## Discussion

The main hypothesis of this study was that health care employees with health complaints who reported favorable physical and psychosocial working conditions adapted their health behavior to include less sickness absence and less sickness presence. Health behavior was conceptualized into four groups. ‘Good health behavior' was defined as low sickness absence and low sickness presence, ‘recovery behavior' as high sickness absence and low sickness presence, ‘risk behavior' as high sickness presence and low sickness absence and ‘poor health behavior' included individuals with both high sickness absence and high sickness presence.

The empirical results of the study confirmed the existence of a general hierarchy between the four different health behaviors such that most favorable work environment factors were associated with ‘good health behavior' and somewhat fewer with ‘recovery behavior,' while no measured favorable work factors were associated with ‘risk behavior' or with ‘poor health behavior.' These results are in line with findings from international systematic reviews and of studies involving mixed occupational groups that have shown that working conditions affect both sickness absence and sickness presence (5, 7–12, 14, 15, 18).

It might seem contradictory that the favorable work environment conditions that were related to ‘recovery behavior' were partly similar to those of ‘good health behavior' as high sickness absence, a criterion of the ‘recovery behavior,' has been found to lead to negative long-term health effects (35). However, sickness absence, particularly shorter periods, may also be seen as a means of recreation or recovery (13, 17, 36). Furthermore, previous studies have shown that sickness presence can be detrimental to subsequent health (11, 14, 18, 22).

Similarities were found between nursing professionals and care assistants regarding the associations between all favorable work environment factors and ‘good health behavior.' There was, however, dissimilarity regarding ‘recovery behavior,' as it was associated with many of the favorable factors among care assistants but associated only with a few favorable factors among nurses. The similarities confirm that working conditions in these health and care working environments are generally similar for the occupational categories studied (3, 26, 37). The differences in terms of the varying effect sizes between the two occupational groups indicate that factors other than those here studied may be associated with ‘recovery behavior' (5, 38, 39).

The study also found that factors measuring positive general work assessments showed high odds ratios for both occupational groups for the associations with both ‘good health behavior' and with ‘recovery behavior.' To the best of our knowledge, no recent study has studied the roles of subjective work assessments and feelings regarding health behavior among health and social care employees. However, a few studies have emphasized the importance of emotional well-being and job satisfaction for understanding work conditions among nursing professionals (13, 40–42).

Many of the associations found between favorable working conditions and ‘good health behavior' and ‘recovery behavior' were weaker or not significant when health status was introduced as a confounder. This was particularly the case among nursing professionals, whereas most associations remained significant among care assistants. This indicates that the degree of health complaints plays a role in the decision between sickness absence and sickness presence. Care assistants with many complaints are more likely than nurses with many complaints to be sickness absent. Why this is the case remains unexplained.

However, the difference between nurses and care assistants may partly be due to a larger number of care assistants compared to nurses in the study, which affects the confidence intervals. It may also be due to unmeasured differences between the groups regarding, for example, employment safety, part-time work, income level, or professional ethics. These are all known factors that may affect an employee's decision to engage in sickness absence or sickness presence (5, 12, 14, 19, 22, 39, 43).

The findings can be understood in different ways. One interpretation is that a large share of the working population is healthy and only suffers from minor health problems that do not limit their work ability and that most work environments are not causing health damages (15, 20, 25). This implies that employees in health and social care, even those with health complaints, who work in situations without physical or psychosocial risk factors, regard their work as not having any health risks or even potentially health promoting and can therefore practice ‘good health behavior.'

Another interpretation is that the results indicate a selection process, whereby individuals who practice ‘good health behavior' are healthier than those in the other behavior categories although they report some health complaints. This selection process is similar to the so called ‘the healthy worker effect' (44). Alternatively, employees who neither practice sickness absence nor sickness presence may not regard their specific ill health symptoms as a restriction for attending work. Individual health and, especially, individuals' perception of their ability to go to work may also affect how they assess the quality of their work environment in the sense that the experience of health symptoms increases the awareness of detrimental working conditions.

### Strengths and Limitations

The major strengths of the present study are that it is based on a large cohort, that the indicators of health and working conditions have been validated, and that these indicators have been used in several previous studies. The fact that the design was cross-sectional restricts any causal interpretations of the associations regarding the different categories of health behavior and working conditions. It should be noted that the different working conditions in the study were treated as singular items rather than as combinations and interaction effects between two or more of the favorable factors may further strengthen the associations.

Also, the ability to control for differences in self-reported degree of health complaints was an advantage as health differences may be as important as work environment differences in explaining health behavior. But this may not have solved the complexity of differences in health. Although all individuals in the study had health complaints, we had no detailed information about the severity or duration of their health complaints or to what degree the health problems limited their ability to work. It is also likely that individuals with poor health have different response patterns to items regarding their working conditions compared to individuals with good health, as there may be a tendency in some cases to seek explanations for their health problem in their work environment.

Another concern is related to limitations in the measurements of sickness absence and sickness presence in this study. Information on sickness absence was available only for cases 15 days or longer, which means that low sickness absence included absence with short duration. This means that individuals with shorter spells of sickness absence are classified together with individuals without any sickness absence in the ‘good health behavior,' which may be misleading and causing confusion. Information on sickness presence was based on the number of occasions, not on the number of days. Although different measures are reasonably correlated, it restricts comparability with studies that have used number of days (45).

## Conclusions

There was an association between employees' work assessments and health behavior. Reporting favorable physical and psychosocial working conditions were especially common among care assistants associated with ‘good health behavior' and ‘recovery behavior' even when health complaints were considered. Thus, nurses and care assistants who work under favorable working conditions are less likely to engage in a combination of high sickness presence and low sickness absence or in a combination of high sickness presence and high sickness absence despite having some health complaints. This implies that reduced sickness presence can be achieved through improved physical and psychosocial working conditions, specifically focusing on reducing heavy physical work and high psychosocial demands. The economic and social costs of sickness absence and sickness presence for involved employees and employers in the health care sectors can be reduced through improved working conditions, but probably also through organizational measures concerning staffing, supportive leadership, and policies for sickness absence and sickness presence. For political decision-makers and public authorities a similar concern for the complexities of work and health and the long-term dangers of both sickness absence and sickness presence should be developed in regulations and supervision.

Longitudinal studies on how favorable working conditions affect the choice between sickness absence and sickness presence at different points in time among health care employees are warranted since they would allow more detailed analyses of the decision processes involved.

## Data Availability Statement

The datasets presented in this article are not readily available because given the restrictions we have from the ethical review board and considering that sensitive personal data are handled, we are not able to make the data freely available. The data may only be provided to other researchers in line with Swedish law and after consultation with the Stockholm University legal department. Requests to access the datasets should be directed to constanze.leineweber@su.se.

## Ethics Statement

The Swedish law on Research Ethics [Swedish Code of Statutes (SFS) 2003:460] states that the use of register data which has been given without consent and contains sensitive information (e.g., regarding health conditions) must receive approval from one of the Regional Research Ethics Committee. This applied to our sickness absence data. Participation in the Work Environment survey had been based on informed consent. Approval must be sought for research use of personal information even when anonymization has taken place after the data linkage. The Regional Ethical Board in Stockholm located at Karolinska Institutet, nowadays part of the Swedish Ethical Review Authority, approved this study (Reference No. 2018/223-31/5) and waived the requirement that informed consent from the research subjects needed to be collected.

## Author Contributions

MH, SM, KG, GA, and CL made substantial contributions to the conception and design of the study. MH did the analyses in collaboration with KG and SM. KG, SM, and MH drafted the manuscript. All authors revised the manuscript critically and approved the final version of the manuscript.

## Conflict of Interest

The authors declare that the research was conducted in the absence of any commercial or financial relationships that could be construed as a potential conflict of interest.

## Abbreviations

SA: Sickness absence
SP: Sickness presence
OR: Odds ratios
CI: Confidence intervals
SWES: Swedish Work Environment Surveys
SCB: Statistics Sweden
LISA: Longitudinal Integrated Database for Health Insurance and Labor Market Studies
SSYK96: Swedish Standard Classification of Occupations 1996
SFS: Swedish Code of Statutes.
