# Supplementary material for: Favorable Working Conditions Related to Health Behavior Among Nurses and Care Assistants in Sweden—A Population-Based Cohort Study
Source: Front Public Health. 2021 Jun 18;9:681971. doi: 10.3389/fpubh.2021.681971 (PMC8249917; doi:10.3389/fpubh.2021.681971)
Supplement: Supplementary file 1 [file Table_1.DOC]

## In the present study, we use 30 questions marked with an *

Answer the following questions by putting an x in the alternative that you best agree with. Put the X

in the middle of the box. If you need to change your answer fill in the entire box and put an X in a new box.

| General questions about your work | | | | | | | | | | | | | | | | |
| --- | --- | --- | --- | --- | --- | --- | --- | --- | --- | --- | --- | --- | --- | --- | --- | --- |
| 1. | In general, are you able to determine your working hours within certain limits?  Yes, I have flexible hours (begin and end work not at exact times, but within certain specified periods )  Yes, I have relatively free working hours in a different way.  No, I am generally unable to affect my working hours. | | | | | | | | | | | | | | | |
| 2. | **Have you staggered working hours, i.e. scheduled shifts with an unpaid break of several hours in the middle of the day?**  Yes  No | | | | | | | | | | | | | | | |
| 3. | How much of your *normal working time* do you usually work at home?  All or nearly all my working time  3-4 days per week  1-2 days per week  A few hours a week  I do not work at home | | | | | | | | | | | | | | | |
| 4. | How easy or difficult would it be for you to get another equivalent job without having to move (change residence)?  Very easy  Rather easy  Rather difficult  Very difficult  Don't know | | | | | | | | | | | | | | | |
| 5. | Are you exposed to any of the following risks or threats in your work? | | | | | | | | | | | | | | | |
|  |  | | | | Yes | | | No | | | | | I am self-employed | | |  |
| a) | Risk that you will be moved to another job against your will | | | |  | | |  | | | | |  | | |
| b) | Threat of termination or forced to work fewer working hours | | | |  | | |  | | | | |  | | |
| 6. | **Do you feel that other working duties are taking too much time from what you are primarily employed to do in your occupation?**  Yes, to a great extent  Yes, to some extent  No  Do not know | | | | | | | | | | | | | | | |
| 7. | Is your immediate supervisor a man or a woman?  Man  Woman  I have no supervisor | | | | | | | | | | | | | | | |
| 8. | We assume that your ability to work, when it was best, is valued at 10 points. How many points would you give your current ability to work? *(Please tick the appropriate number, 0 means that you are unable to work, 10 means that your ability to work is at its best right now.)*   | 0 | 1 | 2 | 3 | 4 | 5 | 6 | 7 | 8 | 9 | 10 | | --- | --- | --- | --- | --- | --- | --- | --- | --- | --- | --- | |  |  |  |  |  |  |  |  |  |  |  | | I cannot work at all |  |  |  |  |  |  |  |  |  | My ability to work is at its best | | | | | | | | | | | | | | | | |
| Stress, requirements, influence and social relationships | | | | | | | | | | | | | | | | |
|  | |  | | | | | | | | | | | | | | |
|  | |  | Nearly  all the  time | About  3/4 of  the time | | Half  the time | | | | About  1/4 of  the time | | About  1/10 of  the time | | | No, not  at all | |
| **9.*** | | **Do you have the opportunity to determine your work pace?** |  |  | |  | | | |  | |  | | |  | |
| **10. *** | | **Is your work so stressful that you do not have time to talk or even think about something other than work?** |  |  | |  | | | |  | |  | | |  | |
| **11.** | | **Can you take short breaks at virtually any time?** |  |  | |  | | | |  | |  | | |  | |
| **12.** * | | **Does the work require your full attention and concentration?** |  |  | |  | | | |  | |  | | |  | |
|  | |  |  |  | |  | | | |  | |  | | |  | |
|  | |  | | | | | | | | | | | | | | |
|  | |  |  |  | | Always | | | | Mostly | | Mostly  not | | | Never | |
| **13.** | | **If your working duties seem difficult, are you able to get advice or help?** | | | |  | | | |  | |  | | |  | |
| **14.** * | | **Are you able to get support and encouragement from *supervisors,* when work feels difficult?** | | | |  | | | |  | |  | | |  | |
| **15.** * | | **Are you able to get support and encouragement from colleagues when work feels difficult?** | | | |  | | | |  | |  | | |  | |
| **16.** | | **If you feel you have too much work to do, can you then get** **information from your supervisor/manager about what to *prioritise?*** | | | |  | | | |  | |  | | |  | |
|  | |  |  |  | |  | | | |  | |  | | |  | |
|  | |  | | | | | | | | | | | | | | |
|  | |  |  |  | | |  | | Yes | | Yes, partly | | | No | | |
| **17** | | **Is it clear what your employer expects you to achieve in your work?** | | | | |  | |  | |  | | |  | | |
| **18.** | | **Does your employer expect that you can be reached by phone during your leisure time?** | | | | |  | |  | |  | | |  | | |
| **19.** | | **Does your employer expect you to read job-related  e-mail during your leisure time?** | | | | |  | |  | |  | | |  | | |

|  |  | | | | | | | | | | | | | |
| --- | --- | --- | --- | --- | --- | --- | --- | --- | --- | --- | --- | --- | --- | --- |
|  |  | | | | Every  day | | A couple  days per  week  (1 day  of 2) | | One day  per  week  (1 day  of 5) | | A couple  days per  month  (1 day  of 10) | | Not at all/  rarely in the  last  3 months | |
| **20.** * | **Do you have so much work that you must miss lunch, work late or take work home?** | | | |  | |  | |  | |  | |  | |
| **21.** | **Does your *supervisor* show appreciation for something you did?** | | | |  | |  | |  | |  | |  | |
| **22.** | **Do *other persons* show appreciation for something you did** (e.g. colleagues, patients, customers, clients, passengers, students)**?** | | | |  | |  | |  | |  | |  | |
| **23.** | **Do you sometimes come in close contact through your work with severely ill people or people with severe problems?** | | | |  | |  | |  | |  | |  | |
|  |  | |  | |  | |  | |  | |  | |  | |
|  |  | | | | | | | | | | | | | |
|  | **Are you involved in any kind of conflict or arguments in your workplace with...** | Every  day | | A couple  days per  week  (1 day  of 2) | | One day  per  week  (1 day  of 5) | | A couple  days per  month  (1 day  of 10) | | A few  times  in the last  3 months | | A few times  in the  last 12  months | | Not at all  in the last  12  months |
| 24. | ...supervisors? |  | |  | |  | |  | |  | |  | |  |
| 25. | ...colleagues? |  | |  | |  | |  | |  | |  | |  |
| 26. | ...other persons (e.g. patients, customers, clients, passengers, students)? |  | |  | |  | |  | |  | |  | |  |
| **27.** | **Are you exposed to violence or threats of violence in your work?** |  | |  | |  | |  | |  | |  | |  |
| **28.** | **Are you subjected to personal harassment by means of malicious words and actions from *supervisors* or *colleagues*?** |  | |  | |  | |  | |  | |  | |  |
|  | In the following two questions, sexual harassment refers to unwanted advances or offensive remarks generally associated with sex. | | | | | | | | | | | | | |
|  | **Are you subjected to sexual harassment in your workplace from...** | Every  day | | A couple  days per  week  (1 day  of 2) | | One day  per  week  (1 day  of 5) | | A couple  days per  month  (1 day  of 10) | | A few  times  in the last  3 months | | A few times in the  last 12  months | | Not at all  in the last  12  months |
| 29. | ...*supervisors* or *colleagues?* |  | |  | |  | |  | |  | |  | |  |
| 30. | ...*other persons* (e.g. customers, patients, clients, passengers, students)? |  | |  | |  | |  | |  | |  | |  |
|  |  | |  | |  | |  | |  | |  | |  | |

| 31. | Do you feel that you are subjected to discrimination in your workplace because of … | | | | | | | | | | | | | | | | | | |
| --- | --- | --- | --- | --- | --- | --- | --- | --- | --- | --- | --- | --- | --- | --- | --- | --- | --- | --- | --- |
|  |  | Yes | | | | No | | | | | | | | | | | | | |
| a) | …sex? |  | | | |  | | | | | | | | | | | | | |
| B) | …sex identity or gender expression? |  | | | |  | | | | | | | | | | | | | |
| c) | …ethnicity? |  | | | |  | | | | | | | | | | | | | |
| d) | …religion or faith? |  | | | |  | | | | | | | | | | | | | |
| e) | …disability? |  | | | |  | | | | | | | | | | | | | |
| f) | …sexual orientation? |  | | | |  | | | | | | | | | | | | | |
| g) | …age? |  | | | |  | | | | | | | | | | | | | |
|  |  | |  | |  | | | |  | | |  | | |  | |  | | |
|  |  | | | | | | | | | | | | | | | | | | |
|  |  | | | Nearly  all the  time | | | | About  3/4 of  the time | | | Half  the time | | | About  1/4 of  the time | | About  1/10 of  the time | | | No, not  at all |
| **32.** | **Are you involved with people who are not employees in the workplace** (e.g. patients, customers, clients, passengers, students, etc.)**?** | | |  | | | |  | | |  | | |  | |  | | |  |
| **33.** | **Does your work require that you simply repeat the same tasks many times an hour?** | | |  | | | |  | | |  | | |  | |  | | |  |
| **34.** | **Does part of your working time involve understanding or solving complicated problems?** | | |  | | | |  | | |  | | |  | |  | | |  |
|  | | | | | | | | | | | | | | | | | | | |
|  |  | | | | | | | | | | | | | | | | | | |
|  |  | | | | | | | | | Always | | | | Mostly | | Mostly  not | | | No, not  at all |
| **35.** * | **Are you able to determine when various working duties are to be carried out** (for example, by choosing to work a bit faster on some days and taking it easier on other days)**?** | | | | | | | | |  | | | |  | |  | | |  |
| **36.** * | **Do you participate in decisions on the arrangement of your work** (e.g. what is to be done, how to do it or who will work with you)**?** | | | | | | | | |  | | | |  | |  | | |  |
|  |  | | | | | | | | | | | | | | | | | | |
| Your situation during the last three months | | | | | | | | | | | | | | | | | | | |
|  |  | | | | | | | | | | | | | | | | | | |
|  | **Have you during the past three months after  work had pain in...** | | | | | | Every  day | | | A couple  days per  week  (1 day  of 2) | | | One day  per  week  (1 day  of 5) | | | A couple  days per  month  (1 day  of 10) | | Not at all/  rarely in the last  3 months | |
| 37. * | ...upper back or neck? | | | | | |  | | |  | | |  | | |  | |  | |
| 38. * | ...lower back? | | | | | |  | | |  | | |  | | |  | |  | |
| 39. * | ...shoulders or arms? | | | | | |  | | |  | | |  | | |  | |  | |
| 40. * | ...wrists or hands? | | | | | |  | | |  | | |  | | |  | |  | |
| 41. | ...hips, legs, knees or feet? | | | | | |  | | |  | | |  | | |  | |  | |

|  |  | | | | | |
| --- | --- | --- | --- | --- | --- | --- |
|  |  | Every  day | A couple  days per  week  (1 day  of 2) | One day  per  week  (1 day  of 5) | A couple  days per  month  (1 day  of 10) | Not at all/  rarely in the last  3 months |
| **42.** * | **When you come home from work  do you feel physically tired?** |  |  |  |  |  |
| **43.** | **Do you find that you cannot stop thinking about work when you are free?** |  |  |  |  |  |
|  | | | | | | |
|  |  | | | | | |
|  | **Have you during the last three months...** | Every  day | A couple  days per  week  (1 day  of 2) | One day  per  week  (1 day  of 5) | A couple  days per  month  (1 day  of 10) | Not at all/  rarely in the last  3 months |
| 44. * | ...been tired and listless? |  |  |  |  |  |
| 45. * | ...had a headache? |  |  |  |  |  |
| 46. * | ...had itchy or otherwise irritated eyes? |  |  |  |  |  |
| 47. * | ...had trouble sleeping? |  |  |  |  |  |
|  | | | | | | |
|  |  | | | | | |
|  |  | Every  day | A couple  days per  week  (1 day  of 2) | One day  per  week  (1 day  of 5) | A couple  days per  month  (1 day  of 10) | Not at all/  rarely in the last  3 months |
| **48.** * | **Do you feel anxiety when you go to work?** |  |  |  |  |  |
| **49.** * | **Do you feel ill at ease and downhearted as a result of the difficulties you face at work?** |  |  |  |  |  |
| **50.** * | **At the end of your work day, do you feel that your work input is inadequate?** |  |  |  |  |  |
| **51.** | **Following work, are you too tired or do not have time for family, friends or leisure activities?** |  |  |  |  |  |
| **52.** | **Do you work alone and are at risk of getting into unsafe or threatening situations?** |  |  |  |  |  |
| **53.** * | **Do you bend or twist yourself in your work in the same way repeatedly in an hour, for several hours during the same day?** |  |  |  |  |  |
| **54.** * | **Are you required to lift at least 15 kg at a time several times per day?** |  |  |  |  |  |

| Working postures and movements | | | | | | | |
| --- | --- | --- | --- | --- | --- | --- | --- |
|  |  | | | | | | |
|  |  | Nearly  all the  time | About  3/4 of  the time | Half  the time | About  1/4 of  the time | About  1/10 of  the time | No, not  at all |
| **55.** * | **Does your job mean that you work is purely physical, i.e. do you put in more physical effort than you do when you walk, stand and move in the usual way?** |  |  |  |  |  |  |
| **56.** * | **Do you exert yourself so much that you breathe faster?** |  |  |  |  |  |  |
| **57.** * | **Do you work bent forward, without supporting yourself with your hands or arms?** |  |  |  |  |  |  |
| **58.** * | **Do you work in a twisted position?** |  |  |  |  |  |  |
| **59.** | **Do you work with your hands raised at shoulder height or above?** |  |  |  |  |  |  |
| **60.** | **Do you work in a sitting position?** |  |  |  |  |  |  |
|  | | | | | | | |
| 61. | What is the longest time you usually sit *without a break* during a typical workday?  More than two hours  Between 1 and 2 hours  Maximum one hour | | | | | | |
| 62. | Do you use a car in your work?  *Does not refer to the trip between home and work.*  Yes, but not as a professional driver  Yes, as professional driver (trucks, taxis, emergency vehicles, etc.)  No  **IF YES** | | | | | | |
| **63.** | **How much of your working time is spent in the car?** | Nearly  all the  time | About  3/4 of  the time | Half  the time | About  1/4 of  the time | About  1/10 of  the time |  |
|  |  |  |  |  |  |
| Other issues on the physical work environment | | | | | | | |
| 64. | Do you work at least one-fourth of your working time with any of the following machines or equipment? | | | | | | |
|  |  | | | | | Yes | No |
| a) | Hand-held machines/equipment or machines that can be moved | | | | |  |  |
| b) | Mobile machines that, for example, you can sit in and drive, but not bus or car | | | | |  |  |
| c) | Stationary machines or process systems, for example, manufacturing, processing,  transport or packaging | | | | |  |  |

| 65. | **How much of your working time takes place in an office environment?** | Nearly  all the  time | About  3/4 of  the time | Half  the time | About  1/4 of  the time | About  1/10 of  the time | No time at all |
| --- | --- | --- | --- | --- | --- | --- | --- |
|  |  |  |  |  |  | *Go to ques-tion 70* | *Go to ques-tion 70* |
| **66.** | **What kind of workplace do you have when you work in an office environment?** *Mark only one answer*  Own room  Own desk in a room for 2-3 people  Own desk in a room for 4-9 people  Own desk in a room for 10-24 people  Own desk in a room for at least 25 people  Borrow someone else's desk that is free  May choose among non-designated desks/workstations  Another type of workplace | | | | | | |
| **67.** | **Do you have access to a room at the office for undisturbed work, phone calls, less spontaneous meetings, etc.?**  Yes, sufficient access  Yes, but access is insufficient  No, not at all | | | | | | |
| **68.** | **Do you have a height-adjustable desk at the office that makes it easy to switch between sitting and standing working positions?**  Yes  No  It depends on which workstation I have | | | | | | |
| **69.** | **Do you find that the room/premises where you work enable you to do a good job?**  To a great extent  Quite a lot  To some extent  Not at all | | | | | | |
|  | **Are you exposed to any of the following in your work?** | Nearly  all the  time | About  3/4 of  the time | Half  the time | About  1/4 of  the time | About  1/10 of  the time | No, not  at all |
| 70. | Noise that is so loud that you cannot converse in a normal tone. |  |  |  |  |  |  |
| 71. | Vibrations, which cause *the whole body to shake and vibrate* (e.g. from a tractor, forklift or other machinery). |  |  |  |  |  |  |
| 72. | Vibrations from hand-held machinery or tools. |  |  |  |  |  |  |
| 73. | Cold (outdoor work in winter, work in refrigerated rooms or similar). |  |  |  |  |  |  |
| 74. | Poor lighting (too weak/glare). |  |  |  |  |  |  |
| 75. | Oil or cutting fluids (in contact with the skin). |  |  |  |  |  |  |

|  | **Are you exposed to any of the following in your work?** | Nearly  all the  time | About  3/4 of  the time | Half  the time | About  1/4 of  the time | About  1/10 of  the time | | No, not  at all |
| --- | --- | --- | --- | --- | --- | --- | --- | --- |
| 76. | Contact with acidic or alkaline (caustic) chemicals. |  |  |  |  |  | |  |
| 77. * | Detergents and/or disinfectants (in contact with the skin). |  |  |  |  |  | |  |
| 78. * | Water that comes in direct contact with the skin several times per hour (including when washing) |  |  |  |  |  | |  |
| 79. * | Human secretions such as saliva, blood, urine, faeces or vomit. |  |  |  |  |  | |  |
|  | | | | | | | | |
|  | **Can any of the following be seen in the air or smelled at your workplace?** | Nearly  all the  time | About  3/4 of  the time | Half  the time | About  1/4 of  the time | About  1/10 of  the time | | No, not  at all |
| 80. | Dust from metals, stone, quartz, cement, asbestos, mineral wool or similar. |  |  |  |  |  | |  |
| 81. | Dust from textiles, wood, flour, animals or plants. |  |  |  |  |  | |  |
| 82. | Chemicals (gases, vapours, solvents, pesticides or plastics chemicals). |  |  |  |  |  | |  |
| 83. | Tobacco smoke from other people's smoking (passive smoking). |  |  |  |  |  | |  |
|  | | | | | | | | |
| Education and training | | | | | | | | |
|  |  | | | | | | | |
|  |  | | Every  day | A couple  days per  week  (1 day  of 2) | One day  per  week  (1 day  of 5) | A couple  days per  month  (1 day  of 10) | Not at all/  rarely in the last  3 months | |
| **84.** | **Does your work enable you to learn something new and develop in your occupation?** | |  |  |  |  |  | |
|  | | | | | | | | |
| 85. | Have you during the past 12 months received training during paid working hours?  Yes, less than 2 days  Yes, 2-4 days  Yes, 5-9 days  Yes, 10 days or more  No | | | | | | | |
| 86. | How do the requirements of your work match your knowledge and skills?  The requirements are much higher than my knowledge and skills  The requirements are slightly higher than my knowledge and skills  The requirements correspond to my knowledge and skills  The requirements are slightly lower than my knowledge and skills  The requirements are much lower than my knowledge and skills | | | | | | | |

| 87. | **Does your job require some learning time or introductory training in the workplace before it can be carried out?**  No  Only a few days  A couple of weeks  A few months  Six months  One year or more | | | | | | | | | | | |
| --- | --- | --- | --- | --- | --- | --- | --- | --- | --- | --- | --- | --- |
| Open or closed atmosphere at work | | | | | | | | | | | | |
|  |  | | | | | | | | | | | |
|  |  | | |  | | Always | | Mostly | | Mostly not | Never | |
| **88.** | **Are you reluctant to express critical views in the workplace regarding your working conditions?** | | |  | |  | |  | |  |  | |
|  | | | | | | | | | | | | |
| Work environment issues | | | | | | | | | | | | |
| 89. | Is it clear who is responsible for work environment issues in your workplace?  Yes  No  Do not know | | | | | | | | | | | |
| **Systematic work environment issues means that:**   - Employers regularly examine working conditions and assess the risks of illness or accidents at work. - The employer immediately or as soon as practicable implements the measures needed to prevent illness and accidents. | | | | | | | | | | | | |
| 90. | Is there ongoing systematic work with work environment issues in your workplace?  Yes  No  Do not know | | | | | | | | | | | |
|  |  | | | | | | | | | | | |
| **91.** | **Have deficiencies in the work environment at your workplace been taken care of within what can be considered reasonable time?** | Always | Mostly | | Mostly  not | | Never | | Not relevant  There are  no work environment deficiencies | | | Do not know |
|  |  | |  | |  | |  | | |  |
|  | | | | | | | | | | | | |
| 92. | Do you have any assignment or related task with work environment issues in your workplace?  Yes, as a safety representative  Yes, as a member of the work environment committee  Yes, as a supervisor  No *Go to question 97* | | | | | | | | | | | |

| Questions about systematic work with work environment issues directed to supervisor,   safety representative or member of the work environment committee | | | | | | |
| --- | --- | --- | --- | --- | --- | --- |
| 93. | Has your company, your management or the equivalent investigated the work environment and assessed the risks of illness and accidents in your workplace?  Yes  Work is now underway  *Safety representative: Go to question 96*  *Others: Go to question 97*  No  Do not know | | | | | |
| 94. | Have the risks noted in the investigation of the work environment according to question 93 above been taken care of?  Yes  Work is now underway  No  Do not know  There were no risks that needed to be taken care of | | | | | |
| 95. | If one or more risks were not taken care of directly, was there an action plan that described when these risks would be taken care of and who is responsible for handling it?  Yes  No  Do not know  There were no remaining risks to take care of | | | | | |
| Question on systematic work on work environment issues directed only towards the safety representative | | | | | | |
|  |  | | | | | |
|  |  | A lot | Quite  a lot | Very  little | Little or  not at all | No  such  work  is  underway |
| **96.** | **As safety representative, do you participate in the systematic work with work environment issues in your workplace?** |  |  |  |  |  |
|  | | | | | | |
| Company healthcare | | | | | | |
| 97. | Do you have access to company healthcare services through your work?  Yes  No *Go to question 102*  Do not know  *Go to question 102* | | | | | |
| 98. | Has anyone from the company healthcare service been at your workplace during the last 12 months? For example, to assess possible risks at the workplace and suggest solutions if necessary?  Yes  No  Do not know | | | | | |
| 99. | Has the company healthcare service otherwise made an assessment of your work situation? For example, when you visited the company healthcare service or in connection with a telephone contact.  Yes  No | | | | | |
| 100. | Do you feel that the company healthcare service has a role in the work to improve the working environment in your workplace?  Yes  No  Do not know | | | | | |
| 101. | If you contact the company healthcare service, do you first need the approval of your supervisor?  Yes  No  Do not know | | | | | |
| Disability | | | | | | |
| Disability here refers to a cognitive impairment, such as difficulty concentrating, remembering, reading, writing or expressing oneself. It can include impaired vision, impaired hearing, reduced mobility, hypersensitivity, mental disability/illness, fatigue, pain, or something similar. | | | | | | |
| **102.** | **With this definition, do you have a disability?**  Yes  No *Go to question 106* | | | | | |
| **103.** | **Do you consider that your disability results in a reduced ability to work?**  Yes  No  *Go to question 106* | | | | | |
| **104.** | **Does your employer know that you have a disability?**  Yes  No *Go to question 106* | | | | | |
| **105.** | **Do you think that the employer has done what is possible to facilitate your work?**  Yes, to a great extent  Yes, to some extent  No, not at all | | | | | |

| Time for recovery | | | | | | | | | | | | | | |
| --- | --- | --- | --- | --- | --- | --- | --- | --- | --- | --- | --- | --- | --- | --- |
|  |  | | | | | | Yes,  Definite-ly  Suffi-  cient | | Yes, mostly  Suffi-cient | | No,  Some-what  Insuffi-cient | No, clearly  Insuffi-cient | | No, far  from sufficient |
| **106.** | **Do you think that you get enough sleep?** | | | | | |  | |  | |  |  | |  |
| **107.** | **In addition to sleep, do you feel that you get enough rest/relaxation between work days?** | | | | | |  | |  | |  |  | |  |
| Work and illness | | | | | | | | | | | | | | |
| 108. * | **How many times during the past 12 months have you worked, even though you really should have not worked given your medical condition?**  Never *Go to question 110*  Once  Two to three times  Four times or more | | | | | | | | | | | | | |
| **109.** | **For what reason or reasons have you worked although you were sick?** *Select one or more options*  No one else can do the job  I cannot afford to be sick  I enjoy my work  I do not want to be considered lazy or unproductive  I do not want to burden my colleagues  I'm afraid of losing work  Working is good for health  I want to stay in touch with colleagues  I am proud of not being sick  Other reasons | | | | | | | | | | | | | |
|  | | | | | | | | | | | | | | |
| Changing working duties for health reasons | | | | | | | | | | | | | | |
| The following questions are about you having changed your work situation or having considered (seriously considered) doing so for *health reasons* during the last 12 months. Other reasons for changing your working duties are not included. (Then respond "No“.) Answer each question. | | | | | | | | | | | | | | |
| 110. | **Have you because of *health reasons* in the last year considered…**  ...changing working duties? | | | | Yes, I have considered it | | | | | Yes, and I have made the change | | | No | |
|  | | | | |  | | |  | |
| 111. | ...changing current working duties? | | | |  | | | | |  | | |  | |
| 112. | ...changing employer or becoming self-employed? | | | |  | | | | |  | | |  | |
| 113. | ...reducing working hours? | | | |  | | | | |  | | |  | |
|  | | | | | | | | | | | | | | |
| 114. | *If you made any change according to the above; if not, please go to question 115.*  Has the change resulted in any improvement of the conditions in your work environment that affected your health negatively?  Yes, very much  Yes, somewhat  No  Do not know | | | | | | | | | | | | | |
| Your experiences with your work | | | | | | | | | | | | | | |
| **How do you experience your work?**  Describe how it usually feels for you by putting an X on each line. The further to the left you put your X, the more you agree with the statement to the left. The further to the right you put your X, the more you agree with the statement to the right. Next furthest out means that you only partly agree/disagree. | | | | | | | | | | | | | | |
|  |  | Neither  Nor | | | | | | | |  | | | | |
|  |  | 1 | 2 | 3 | | 4 | | 5 | |  | | | | |
| 115. | Far too much to do |  |  |  | |  | |  | | Far too little to do | | | | |
| 116. | Far too difficult working duties |  |  |  | |  | |  | | Far too simple working duties | | | | |
| 117. | Too little influence |  |  |  | |  | |  | | Too much influence | | | | |
| 118. | Too little contact with others |  |  |  | |  | |  | | Too much contact with others | | | | |
| 119. | Mentally demanding work |  |  |  | |  | |  | | Mentally light work | | | | |
| 120. | Monotonous work |  |  |  | |  | |  | | Varied work | | | | |
| 121. | Restricted and non-free |  |  |  | |  | |  | | Unrestricted and free | | | | |
| 122. | Very dissatisfied with my working hours |  |  |  | |  | |  | | Very satisfied with my working hours | | | | |
| 123. | Very meaningless work |  |  |  | |  | |  | | Very meaningful work | | | | |
| 124. | Physically demanding work |  |  |  | |  | |  | | Physically light work | | | | |
| 125. | Strenuous working postures |  |  |  | |  | |  | | Comfortable working postures | | | | |
| 126. | Overall, I am dissatisfied with my work |  |  |  | |  | |  | | Overall, I am satisfied  with my work | | | | |

**Thank you for your cooperation!**
